# Contextual behavior and neural circuits

**DOI:** 10.3389/fncir.2013.00084

**Published:** 2013-05-10

**Authors:** Inah Lee, Choong-Hee Lee

**Affiliations:** Behavioral Neurophysiology Laboratory, Department of Brain and Cognitive Sciences, Seoul National UniversitySeoul, South Korea

**Keywords:** hippocampus, context, perirhinal cortex, post-rhinal cortex, entorhinal cortex, response selection, choice behavior, decision making

## Abstract

Animals including humans engage in goal-directed behavior flexibly in response to items and their background, which is called contextual behavior in this review. Although the concept of context has long been studied, there are differences among researchers in defining and experimenting with the concept. The current review aims to provide a categorical framework within which not only the neural mechanisms of contextual information processing but also the contextual behavior can be studied in more concrete ways. For this purpose, we categorize contextual behavior into three subcategories as follows by considering the types of interactions among context, item, and response: *contextual response selection, contextual item selection*, and *contextual item–response selection*. Contextual response selection refers to the animal emitting different types of responses to the same item depending on the context in the background. Contextual item selection occurs when there are multiple items that need to be chosen in a contextual manner. Finally, when multiple items and multiple contexts are involved, contextual item–response selection takes place whereby the animal either chooses an item or inhibits such a response depending on item–context paired association. The literature suggests that the rhinal cortical regions and the hippocampal formation play key roles in mnemonically categorizing and recognizing contextual representations and the associated items. In addition, it appears that the fronto-striatal cortical loops in connection with the contextual information-processing areas critically control the flexible deployment of adaptive action sets and motor responses for maximizing goals. We suggest that contextual information processing should be investigated in experimental settings where contextual stimuli and resulting behaviors are clearly defined and measurable, considering the dynamic top-down and bottom-up interactions among the neural systems for contextual behavior.

## Context and contextual behavior

**Figure 1:**
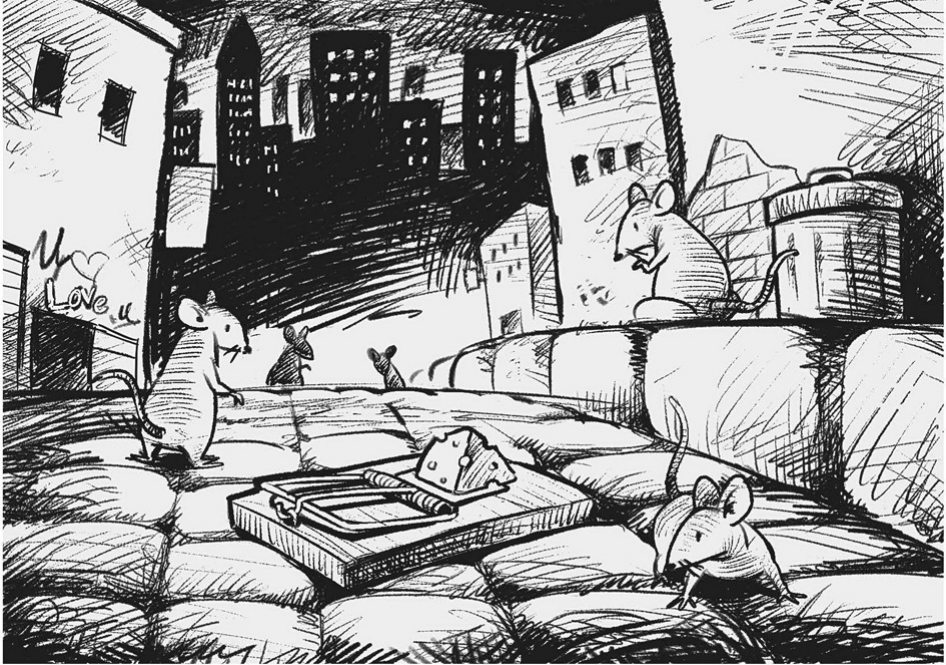
**A cartoon for illustrating the importance of contextual interpretation of items for choice behavior.** Rodents usually avoid novel objects (cheese over the trap in this example) paired with a surrounding context to stay away from danger.

Animals including humans recognize their surrounding environment rapidly and with seeming ease. A surrounding environment often influences which information needs to be attentively processed in certain ways and also determines an appropriate action set or behavioral repertoire (Figure 1). That is, the surrounding environment often determines the *modus operandi* of the brain. In the absence of such powerful information that effectively sets up the global mode of operation in the brain, physical stimuli in the ever-changing and complex world should produce insurmountable ambiguity and confusion in neural information processing and resulting behavior. In the current review, we will selectively review prior studies including our own that can speak to how the brain enables an animal to learn and remember to respond appropriately in a contextual manner.

## Definition and scope of context and contextual behavior

What is context? There are many contexts one may encounter during lifetime and there are as many definitions of the term as well. *Context* is hardly a scientific term. It is often casually used in layman's conversation to loosely refer to a set of physical and cognitive factors that determine the meaning of an otherwise ambiguous element(s) associated with those factors. When a behavioral response to such an element is influenced by those peripherally related factors, one may call it a contextual behavior. The purpose of the current review is to provide a perspective on how contextual system interacts with different neural systems for response, such as item information-processing systems and motor action systems (see below). Therefore, we would not be able to provide an extensive review of the literature on context, but briefly mentioning some related contents from the literature would nonetheless be helpful.

The concept of contextual stimuli was first brought to learning and memory research as learning theories developed largely within the framework of classical and instrumental conditioning (Rescorla and Wagner, 1972). At that time, context was considered largely as stimuli in the background that can be processed by the same learning principles as for elemental cues (e.g., light, tone, etc.) in an apparatus. A context, in this case, refers to background stimuli that are not directly manipulated in the experiment as opposed to phasic stimuli such as tone and light that serve as a direct predictor of reward. Defining context in this manner could potentially be problematic because the concept is defined by what is not (i.e., foreground phasic stimulus) rather than by what it is. In most cases, a single elemental stimulus (e.g., an object, an odor, a single tone, etc.) presented as a cue in an experiment is not normally called a context. In contrast, a set of many objects, for example, whose individual parts one may not be able to easily and rapidly recognize in the background may be called a context. That is, contextual information is “multiplex” by nature. The threshold that separates the two states (i.e., elemental vs. contextual stimuli), however, may not be clearly defined just by counting the number of elemental stimuli included. Nadel and Wilner thus proposed another important dimension of context that may compensate for this and that was whether a stimulus was phasic or tonic in terms of frequency of change (Nadel and Wilner, 1980; Nadel, 2008).

The above properties of context (i.e., static background composed of multiple elemental cues) are largely associated with the physical nature of stimuli (i.e., multiplexed information content and temporal duration and frequency of occurrence). On the other hand, more functional definitions of context were proposed by several researchers (Hirsh, 1974; Good and Honey, 1991; Myers and Gluck, 1994). Hirsh (1974), for example, proposed for the first time that the hippocampus was involved in retrieving contextual memory. According to Hirsh's theory, an external stimulus (S) elicits a specific response (R) and such S–R association chains are stored and expressed through a so-called “performance line.” For example, when a rat learns to press a bar to obtain reward, the association between the bar and pressing response is stored through a performance line. Hirsh argued that the contextual memory system influenced the information flow in the performance line so that, for example, bar-pressing behavior could become more prominent when the rat performed the task in the same context in which it had been trained, compared to a different context. According to his model, therefore, contextual memory works as a gatekeeper for retrieving S–R memories flexibly in consideration of the surrounding background, and the absence of the hippocampus leaves the animal with fixed and rigid S–R memories only. The hypothesized modulatory function of context during memory retrieval may be considered similar to the “occasion setting” property of a stimulus in classical conditioning literature (Holland, 1985; Yoon et al., 2011). For example, if a tone stimulus leads to reinforcement only when it is preceded by a light stimulus, but not when the tone is presented by itself, then the light sets the “occasion” for reinforcement. When a contextual stimulus in the background functions as a gatekeeper, the context may be called an occasion setter also (Myers and Gluck, 1994; Yoon et al., 2011). However, in this review, we will use the term context instead of occasion setter because the occasion setting hypothesis does not necessarily require the occasion-setting stimulus to be multiplexed as for context. That is, an elemental stimulus may well function as an occasion setter. Since different neural systems are likely to be recruited depending on the nature of the stimulus, this distinction between elemental stimulus and multiplexed stimulus (i.e., context) is important.

Removing the hippocampus may result in rigid and inflexible S–R memory systems, which would be equivalent to having only habit memory systems (Graybiel, 2008). Hippocampal-lesioned animals indeed show perseverative behavior (Whishaw and Tomie, 1997) and this reemphasizes the importance of contextual information. Specifically, one of the biggest problems of having only the rigid S–R memory system would be not being able to deal with ambiguous stimuli. For example, when a particular stimulus results in reward in one context, but not in another environment, the same stimulus now possesses ambivalent or conflicting reward values. In other cases, a stimulus may be presented in an incomplete or noisy form. In these cases, the surrounding background information is vital for resolving the ambiguity associated with the stimulus. That is, a context solves “predictable ambiguity” (Morris, 2007). Once context resolves ambiguity, a more decisive action can be made. Clearing ambiguity for achieving decisiveness and speed also occurs not only on the motor action side, but also in stimulus processing because there should be contextual top-down influence on how individual stimuli in the environment should be processed (in conjunction with relevant motivational and emotional states as well as rules and strategies). Context, therefore, provides a unifying cognitive framework in the brain from stimulus interpretation to behavior.

In the current review, we will define the term context as follows. Context refers to an external physical stimulus that is present in the animal's background and is multiplex (i.e., composed of complex elemental information), and it should be directly associated with purposeful behavior. By restricting the scope of definition to external and physical stimuli, we intend not to include non-environmental internal variables such as rule, strategy, sequence, motivation, drug state, linguistic variables, etc., as examples of context (Oler and Markus, 2000; Bouton, 2002; Kennedy and Shapiro, 2004; Smith and Mizumori, 2006). This is mainly because, as Nadel pointed out in his review (Nadel, 2008), the same internal states (e.g., hunger or thirst) can often be associated with different (sometimes orthogonal) external environments and this makes the concept of context unnecessarily complex and fuzzy. Good and Honey, showed that hippocampal lesioned subjects were impaired in learning that a given elemental stimulus was differentially associated with reinforcement in different contexts (Skinner boxes housed in different rooms with different odors and wallpapers) (Good and Honey, 1991). The example of context in the Good and Honey study can be defined strictly based on physical stimuli in the background and may well fit the definition of context in this review. We do not, however, presuppose that a context should be always multimodal. A context should be multiplex in nature; that is, it should be composed of multiple elemental cues but may not be easily decomposable into elemental cues, and it should thus be fairly complex and cohesive. This also relates to the functional definition and that is, for a well-constructed contextual stimulus, learning to respond in a particular way in association with a single element in the context should lead to suboptimal or counterproductive results in most cases. For example, when the rat is trained to use multiple external cues in the distal background for retrieving a spatial location, the multiple distal cues can be considered as context and normal rats can effectively retrieve location memory when some elemental cues in the context are missing (Gold and Kesner, 2005). However, rats with hippocampal lesions are markedly impaired as the distal background loses some elemental cues, suggesting that the rats without the hippocampi use individual cues instead of using the entire cues as a whole as a context. In other words, contextual effects on behavior should be maximal when the contextual stimulus is processed as a Gestalt rather than as a linear sum of elemental stimuli. Fanselow (1986) has noted that it takes time to develop a contextual representation because of this nature as opposed to elemental stimuli. The constraint that a context should remain in the background is important as Smith defined a context “that which surrounds” (Smith, 2007). Remaining in the background can operationally be defined as the contextual stimulus not becoming the direct target of response. By this definition, therefore, when the rat swims toward a large patterned visual cue in a modified water maze to reach the platform placed directly underneath the cue (Prusky et al., 2004), such visual cues will not be considered as a context in this review because the visual cue in that case is a target stimulus itself and does not serve as a background. Using visual patterns in this way (i.e., foreground target for response) also does not powerfully recruit the hippocampus either (Kim et al., 2012; Lee and Lee, 2012). We do not necessarily require a context to be completely static because the surrounding background perceptually changes all the time in real life to varying degrees especially when the animal moves across different environments. Although the hippocampus is involved in the tasks that include aforementioned contextual stimuli as critical components (O'Keefe and Nadel, 1978; Good and Honey, 1991; Penick and Solomon, 1991; Kim and Fanselow, 1992; Phillips and Ledoux, 1992; Honey and Good, 1993), one should not fall into a circular argument by judging whether a particular stimulus is contextual or not just based on its dependence on the hippocampus.

With respect to contextual behavior, we will selectively review the results from goal-directed tasks only in which a purposeful behavioral response toward a goal (e.g., seeking for reward or avoiding danger) is clearly predefined in relation to context (Lee and Lee, 2012). Studies that used other behavioral paradigms in which animals were allowed to freely forage for food (Muller et al., 1987; Skaggs and McNaughton, 1998; Lee et al., 2004b; Leutgeb et al., 2004) or spontaneously explore objects (Save et al., 1992; Ennaceur et al., 1997; Vazdarjanova and Guzowski, 2004) thus will not be covered in this review for the reasons provided elsewhere (Lee and Lee, 2012).

## Categories of contextual behavior and brain regions

**Figure 2:**
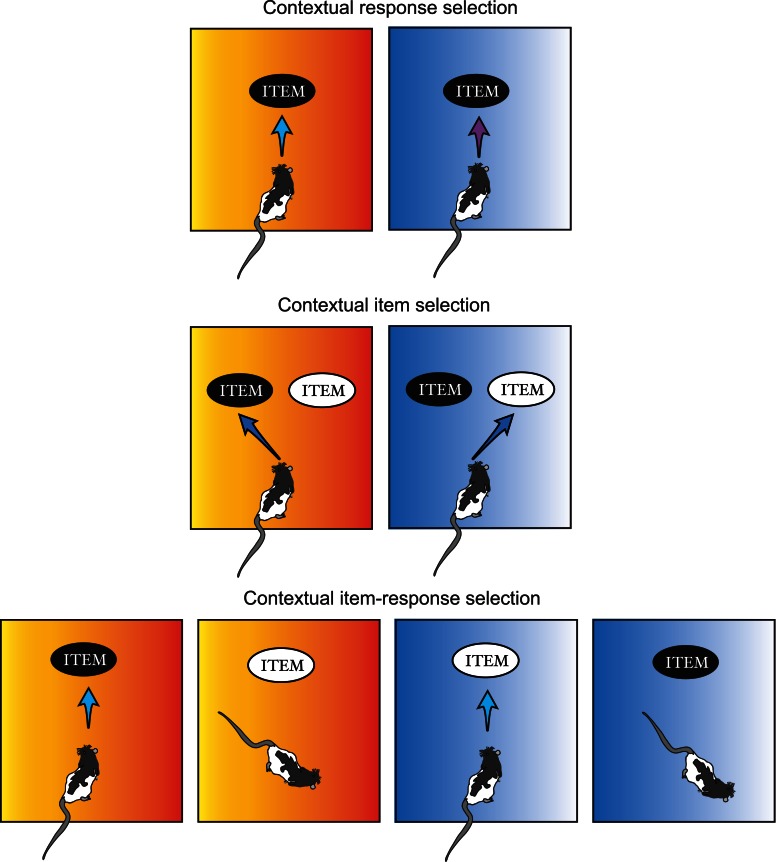
**Categorization of contextual behavior.** Contextual behaviors are grouped into three categories in this review depending on the relationships among context, item, and the animal's response pattern to the combination of the two. A surrounding context is symbolized as the color-gradient background here. *Contextual response selection*: an animal produces different types of responses (e.g., pushing vs. digging) to the same item depending on which context is associated with the item. *Contextual item selection*: an animal gives out the same type of responses to different items depending on the contextual information. *Contextual item–response selection*: an animal responds to an item (e.g., black item) when it is identified in one context (e.g., orange context), but not when it is encountered in another context (e.g., blue context), and *vice versa* for the other item (e.g., white item).

As described above while selectively overviewing the literature on contextual memory, the modulatory effect of context has been tested traditionally by measuring changes in behavioral response across different contexts. Such experimental paradigms thus test not only how contextual information is represented in memory but also how the represented contextual information is “utilized” during action selection. Although it is likely that the same contextual information recruits different brain regions depending on the type of response required (e.g., freezing, lever pressing, etc.), literature has been focusing heavily on how contextual representation is formed and stored in the brain with less emphasis on how contextual memory is “used” during behavioral choice. We would argue in this review that a more wholistic investigation of neural circuits for contextual behavior requires understanding not only how contextual memory is formed and retrieved, but also how contextual information influences different types of choice behavior. Therefore, contextual behavioral studies will be subdivided into three categories in this review using the number of items and the type of responses associated with multiple contexts as major criteria: *contextual response selection, contextual item selection*, and *contextual item–response selection* (Figure 2 and Table 1). In the first category (contextual response selection), an item in the environment is fixed but different response types toward the item must be contextually determined. In the contextual item-selection category, response type is fixed but the same response should be targeted toward different items depending on which context is associated with the items. The third category is the mixture of both.

**Table 1:** **Categorization of contextual behavior**.

	**Contextual response selection**	**Contextual item selection**	**Contextual item–response selection**
Target item(s)	Fixed	Variable	Variable
Contextual response type(s)	Variable	Fixed	Variable

### Contextual response selection

Contextual response selection is required when an animal should choose a particular behavioral response from a set of learned behavioral choices in association with the environmental context. This often occurs when there is no obvious elemental cue in the environment that reliably tells the animal which behavioral choice is appropriate or when the elemental cue present is ambiguous (thus rendering decision making for choice behavior difficult), but the surrounding context does the job.

#### Rodent studies

One of the widely spread contextual behavioral testing paradigm, contextual freezing or fear-conditioning, requires contextual response selection. In a typical contextual fear-conditioning experiment (Kim and Fanselow, 1992; Phillips and Ledoux, 1992), a rat is put in an operant conditioning chamber from which the animal can look at the surrounding visual context (sometimes in addition to odor and sound stimuli in the chamber). After a few minutes of exploration in the chamber, the rat is electrically shocked through its feet. The aversive experience is then associated with the specific context in which the conditioning chamber was placed because the rat freezes (the usual behavioral measure for testing whether the shock-context association has occurred or not) when placed in the chamber in the same context but not when placed in a different context. Here, response selection occurs between non-freezing/locomotive and freezing behaviors depending on the contextual stimuli in the background. The behavior is conditioned to context rather than to elemental stimuli because the rat freezes even when some of the elemental cues in the environment are removed (Gonzalez et al., 2003). Also, when the rat is not allowed to scan the room context for a proper amount of time presumably for building a contextual representation of the environment, it fails to show context–shock association (Fanselow, 2000). The contextual fear-conditioning paradigm is a good example in which a context is used directly as a conditioning cue for behavior as compared to a modulator for another stimulus–response association (see below in contextual item–response selection). One thing that is peculiar about the contextual fear-conditioning paradigm (compared to other contextual behaviors; see below) is that the behavioral response (i.e., freezing) is not targeted to any item in the environment because it is presumably a self-defensive behavior (Fanselow, 1994). It is speculated that the goals of manifesting freezing behavior are to avoid the movement-sensitive visual systems of predators but the exact utility of this behavior may still need to be experimentally verified for this paradigm to be surely categorized as a goal-directed memory task. Besides the contextual freezing paradigm, behavioral tasks in which rodents make discrete spatial choices using a surrounding visual context can also be considered testing contextual response selection. In an 8-arm radial maze, for example, a rat may need to decide which arm to enter on the basis of the distal visual contexts associated with individual arms (Tolman et al., 1946; Olton et al., 1982; Jarrard, 1983; O'Keefe and Speakman, 1987; Lee and Kesner, 2003; Lee and Solivan, 2008; Jo and Lee, 2010a). As in the contextual fear conditioning, visual context is directly associated with the animal's response selection (spatial response in this case as opposed to freezing).

**Figure 3:**
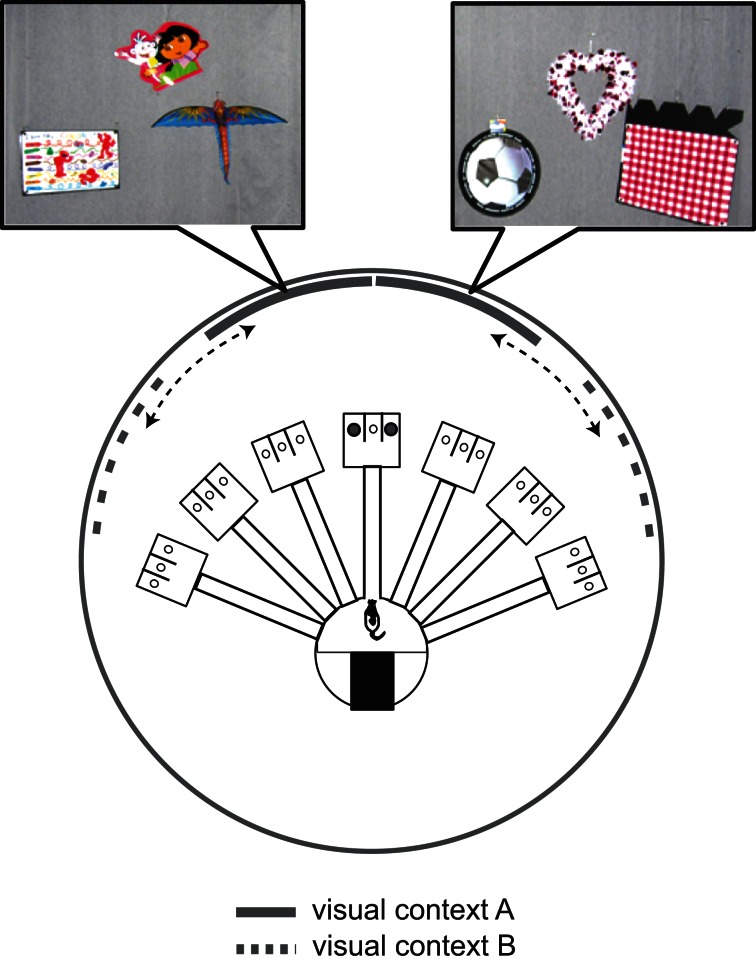
**A contextual response-selection task.** Visual context is defined by the configuration (parameterized by the angular distance) of the two curtains (each with a distinct set of visual cues as shown in the pictures). For example, context A is defined by the two cue-curtains aligned at the center (thick arc lines) and context B is when the two cue-curtains set apart by a larger degree (dotted arc lines).

One of the caveats of using traditional room cues as visual context is that it is difficult to specify exactly which visual stimuli are fed to the neural circuit for contextual information processing. That is, although phenomenologically salient and established well for behavioral effects, it could become a hurdle in the future to examine how contextual information processing is realized in neural circuits if unspecified cues (from the neural-information-processing point of view) are called a context. As an attempt to improve this situation, we tested previously whether the configuration of specific sets of visual cues can serve as effective contextual stimuli for directing the rat's spatial choice behavior to discrete locations (Kim and Lee, 2011). In a visual contextual response-selection (VCRS) task, the rat ran along the same linear track to choose either left or right food well at the end of the track for reward (Figure 3). The rewarding food well in each trial was defined by the configuration (or angular relationships) between two sets of visual cues (each set attached to the same curtain). A context in this study is defined by the configuration of two visual cue sets distally placed from the animal (which fits our definition of context in this review since the multiplexed stimuli are present in the animal's background in association with purposeful behavior). Context-1, for example, was defined by angular distance of 0° between the two distal cue sets and context-2 was set by separating the two cue sets by 80°. Despite the seemingly simple nature of the task, dorsal hippocampal-lesioned rats were severely impaired in this task compared to controls. Inactivations in the dorsal hippocampus (using GABA-A receptor agonist, muscimol, or MUS) also produced the same results. Since the similarity between visual contexts could be parametrically defined by angular distance in this paradigm, it was possible to examine whether similar, yet novel contexts could be processed effectively on the basis of familiar contextual memories. The dorsal hippocampus was critical for such capabilities (Kim and Lee, 2011).

**Figure 4:**
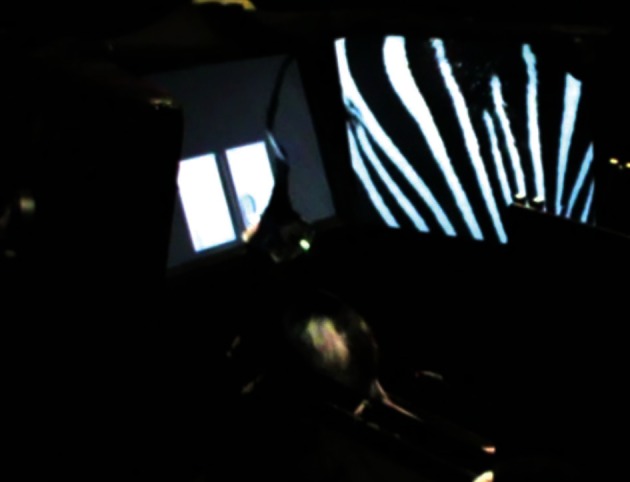
**A touchscreen-based contextual response-selection task.** A side view of the LCD-touchscreen apparatus in which two peripheral LCD screens (only one visible from this particular angle) and the center touchscreen (showing two adjacent response target rectangles) were installed to make the rat to choose a particular response using the surrounding visual context (zebra pattern in this example). The LEDs and tether attached to the rat are for electrophysiological recording.

A more sophisticated version (in terms of stimulus control) of the VCRS task was developed later in our laboratory, using touchscreen and LCD monitors (Kim et al., 2012). Specifically, we designed a task in which two-dimensional patterned visual stimuli were presented in the background via LCD screens (Figure 4). In the task, the rat ran along a linear track and encountered a touchscreen monitor at the end of the track. The rat was required to touch one of the adjacent rectangular box images depending on the visual contextual stimuli displayed in the two peripheral monitors positioned on both sides of the center touchscreen monitor. The rats with MUS infused bilaterally in the dorsal hippocampi showed severe performance deficits in the VCRS task (Kim et al., 2012). The impairment was completely reversible with vehicle injections. Importantly, the impairment in contextual response selection with hippocampal inactivations occurred regardless of whether the visual context was presented in the side monitors or only in the center touchscreen monitor, suggesting that the functional “backgroundness” of large patterned visual stimuli behind the elemental cues (i.e., response box images to which the animal should direct its responses) as well as their roles in resolving ambiguity (Bolles, 1985; Nadel, 2008) in choice behavior are more important in functionally defining a hippocampal-dependent visual context than whether the cues are distally located or not. The hippocampus does not appear to be directly related to perceptually discriminating the visual patterns (Morris et al., 2013) because, when the same visual contextual stimuli were pitted against each other between the two side monitors and as the rats simply ran toward the visual context associated with reward on a T-shaped track, hippocampal inactivations with MUS showed minimal disruptions, if any, in performance (Kim et al., 2012).

**Figure 5:**
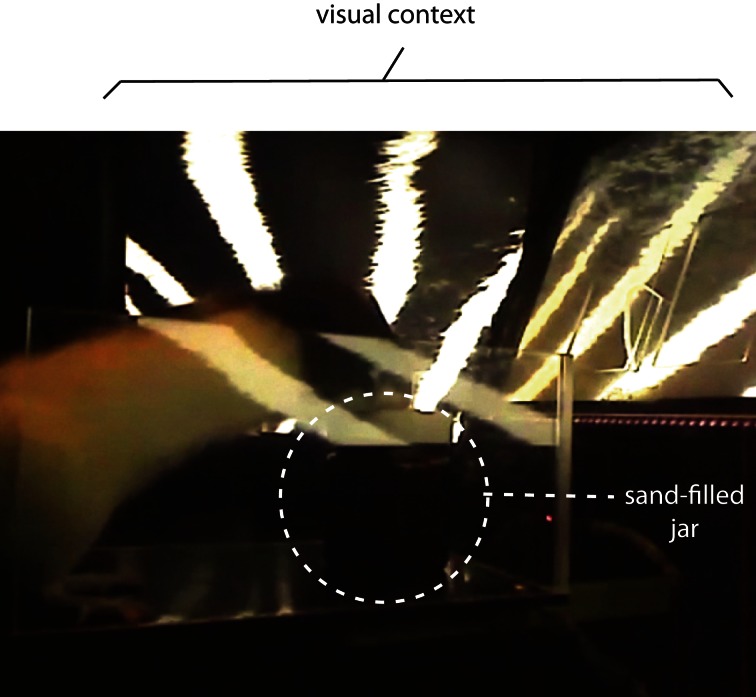
**A contextual response-selection task.** The rat is required to either dig the sand in the sand-filled jar or push the jar to obtain a piece of cereal. The behavior selection is associated with the visual context presented in the background using an array of three LCD monitors (partially shown here).

In a similar paradigm, we also tested the roles of the medial prefrontal cortex (mPFC) recently in another version of VCRS task (Lee and Shin, 2012) in which the animal was required to either push a sand-filled jar or dig the sand in it to obtain a piece of cereal reward according to the visual context presented in the animal's background through an array of LCD monitors (Figure 5). The rat, therefore, must choose between equally plausible responses associated with the same object (sand-filled jar) and only the visual background context determined which response was appropriate in a given context to achieve goals. Inactivations of the mPFC severely disrupted the performance in this task, whereas the same mPFC manipulations failed to impair performance in simply discriminating the two visual contexts, or in performing the same task using elemental cues such as tactile cues instead of visual contexts (Lee and Shin, 2012).

Studies that allow subjects to freely navigate in an open space (Maaswinkel et al., 1999; Day et al., 2003; Hok et al., 2007; Goodrich-Hunsaker et al., 2010) as opposed to the ones that allow only structured movements associated with choice behaviors (e.g., radial arm mazes) may be considered as a special case of contextual response selection in which contexts are continuously monitored and updated and associated responses are also continuously chosen. However, when an animal continuously move around, some movements or behaviors arguably may not be the result of contextual response selection, but may just be bridging behaviors between critical choice behaviors. It may or may not be the case but it certainly makes it difficult to examine the issue when unlimited free navigation is allowed in space. If a cognitive map is composed of multiple contextual representations spatially related to each other (O'Keefe and Nadel, 1978; Nadel, 2008), spatial navigation that allows freeform movements is likely to depend on the cognitive map and its associated path-integrative information and directional signals (Sharp et al., 1996; Redish and Touretzky, 1997; Samsonovich and McNaughton, 1997; Taube, 1998). Discussing such a complex navigation system is beyond the scope of this review and readers should refer to other articles (McNaughton et al., 1996; Redish and Touretzky, 1997; Taube, 1998).

#### Primate and human studies

Compared to rodent experiments, it is more difficult to test visual contextual choice behavior in primates and humans mainly because most experimental paradigms that involve the measurement of brain signals require the subject's head to be fixed in these species (thus limiting the possibility of presenting stimuli in the peripheral background). One of the examples that may be considered closer to a rodent contextual behavioral paradigm would be a study done by Wirth et al. (2003), in which a complex natural visual scene was presented in front of the subject and, after the scene disappeared, required the monkey to remember one of four possible saccadic eye-movement responses (up, down, left, and right) in association with the visual context after a brief delay (700 ms). Although whether viewing a scene in a computer screen is equivalent to viewing surrounding visual cues in the background and whether the task is hippocampal dependent should be investigated further, hippocampal neurons showed neural correlates of learning the scene-response relationships. As in rodent studies using a radial-arm maze, this study thus required the subject to perform behavioral response selection on the basis of a complex visual stimulus with no variable “items” involved as a target of the response (i.e., physical properties of items were fixed).

**Figure 6:**
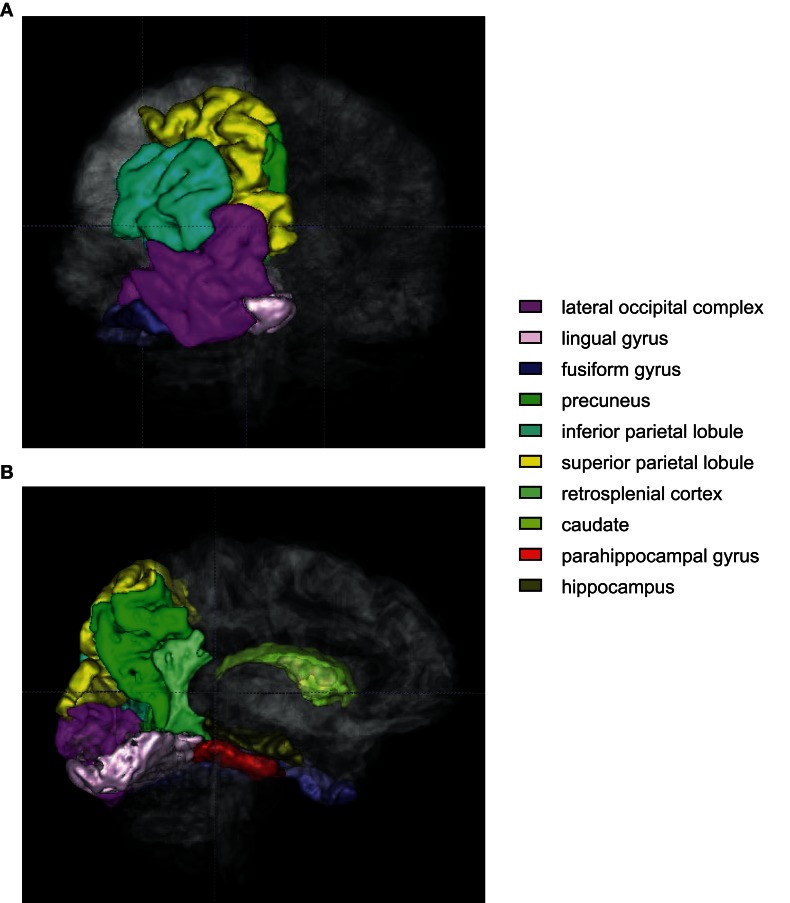
**An illustration of regions of interest in human VR contextual response-selection task.** Human brain regions (color-coded) that are consistently activated in contextual response-selection studies. **(A)** A posterior view of the brain. Regions of interest (left hemisphere only) were overlaid with the translucent whole brain. **(B)** A medial view of the same areas shown in **(A)**. The regions associated with the colors closer to violet are sensory and associational cortex (lateral occipital complex, lingual and fusiform gyri). The regions in green spectrum (inferior/superior parietal lobules, precuneus, retrosplenial cortex, and caudate) are the areas that are also frequently activated in a VR navigation task.

In human studies, combining virtual reality (VR) with functional magnetic resonance imaging (fMRI) techniques has provided a unique experimental opportunity that allows testing visual contextual behavior in a goal-oriented experimental situation that closely resembles the rodent behavioral paradigm (probably in a way that suits the human visual system). Human imaging studies provide unique opportunities to observe how different neural networks in the whole brain change activity patterns simultaneously, which is not currently feasible in rodent studies. In a VR environment, a visual context is provided by realistic graphical background images and the visual background continuously changes as a subject responds to it. In this situation, a subject can perform a contextual response selection by indicating right or left turn, for example, by pushing corresponding buttons or by manipulating a joystick when he/she encounters a particular visual scene or context. Many tasks that may belong to this category used a virtual maze in which egocentric responses were critically required because the subject moved through alleys connected by several intersections. However, some of these tasks (Gron et al., 2000; Weniger and Irle, 2006; Weniger et al., 2010) may not be categorized strictly as for testing contextual response selection. This is because turning decisions were not critically dependent on visual context in the background since contextual visual background was minimized in some experiments and only textured walls of the maze guided the navigation. Navigation in such cases may be more guided by the memories of sequential turns. Nonetheless, the medial occipital gyri, the lingual gyri, the parahippocampal gyri, the medial and lateral superior parietal lobules, and the hippocampus were usually activated in these paradigms (Gron et al., 2000; Weniger and Irle, 2006) as illustrated in Figure 6. Some studies added more contextual information in the alleys or hallways in the maze by putting unique objects in hallways and also making each hallway look distinctively different (Brown et al., 2010, 2012). The task used in the Stern group's studies was a semi-VR environment, however, because a series of still images were presented in response to the subject's choice responses (instead of constantly updated scenes in a true VR environment). The Stern group's studies also made the sequence component of navigation critical by putting an overlapping visual context (i.e., identically looking hallway scene) that needed to be passed by the subject when different navigational routes were traveled. In this situation, once the subject entered the overlapping segment of the route, the subject should use memories of previous paths to make response selection. The hippocampus, parahippocampal cortex, caudate, and orbitofrontal cortex were activated in this situation (Brown et al., 2010, 2012). When a distal visual context was provided in the background in a VR maze environment, navigational strategy-dependent differences were found between the hippocampus (spatial navigators) and striatum (STR) (egocentric navigators) in fMRI signals (Iaria et al., 2003).

The involvement of the posterior parietal lobule and precuneus consistently reported being active during VR navigation tasks is worth mentioning. These regions have not been actively investigated in animal studies especially in rodents (Rogers and Kesner, 2006; Whitlock et al., 2008; Save and Poucet, 2009) compared to other regions in the medial temporal lobe. Zhang and Ekstrom found that the levels of activation in these regions were highly correlated with the retrieval of allocentric spatial information (Zhang and Ekstrom, 2012). In their study, subjects were required to navigate a VR environment in the fMRI scanner in an originally taught way (i.e., fixed landmark-based) or in a different way (i.e., using spatial relationships among the buildings presented in a novel fashion compared to the learning condition). Although the surrounding environment was slightly different from our definition of context in the Zhang and Ekstrom study, it does provide some insights into the roles of the posterior parietal lobule and precuneus. As recently pointed out in a review (Kravitz et al., 2011), it is possible that the posterior parietal region plays key roles when moving through space (egocentrically in most cases as in most human imaging studies in which the allocentric environment moves in relation to the fixed head position of the subject) using allocentric visual contextual information (Rogers and Kesner, 2006; Whitlock et al., 2008; Save and Poucet, 2009). It is also important to note that the areas in the medial temporal lobe (including the hippocampus) are connected with the dorsal visual information-processing stream via the retrosplenial cortex and posterior cingulate cortex (Kravitz et al., 2011). Further studies are needed to understand how the posterior parietal-temporal lobe information streams contribute to contextual behavior.

In VR studies, it is also interesting that the visually impoverished conditions (i.e., poor context) made spatial response selection (e.g., navigation) less efficient and produced some differences in the regions of the brain activated for making behavioral choices as compared to the areas for making responses to contextually rich visual stimuli (Maguire et al., 1998; Rauchs et al., 2008). For example, in addition to the usual hippocampal-cortical networks activated in a VR task such as the hippocampus, lingual gyrus, occipital gyrus, superior parietal gyrus, making behavioral choice in contextually rich environment was more associated with the activities in the fusiform gyrus, superior temporal gyrus, and cuneus (Rauchs et al., 2008). It is interesting that the parahippocampal cortex (in addition to the hippocampus) was more recruited when subjects were required to find an alternate route that deviated from the well-learned route in the Rauchs et al. study.

### Contextual item selection

In comparison to contextual response selection, contextual item selection occurs when an animal needs to direct a single, fixed response to a certain item (among multiple items) in conjunction with a specific context in the animal's background. Context in this case plays a critical role especially when the same item(s) appears in multiple contexts, but is rewarded in a certain context only. Importantly, the same response (e.g., pushing an object) should be emitted in all cases (so devoid of the response-selection requirement). The response in this case just needs to be directed toward the correct item considering the environmental context.

#### Rodent studies

Objects can appear in multiple contexts, but the values attached to those objects may be contextually different. That will make the same response (e.g., pushing) to the same object appropriate sometimes but not in other times. For example, Bussey et al. (2001) tested rats in a double Y-maze in which a pair of objects was presented in one side of the double Y-maze. At the choice point of the Y-track, the rats were required to enter the arm that contained the object associated with reward in a given context. The rats with lesions in the perirhinal cortex (PER) were severely impaired in this task and showed no significant improvement throughout the acquisition period. The PER lesions, however, left the capability of visually discriminating objects intact (Bussey et al., 2001), which suggests that the deficits were not merely based on impairment in visual recognition for objects after lesions. A similar experimental paradigm was also applied in a contextual odor-selection task (Rajji et al., 2006). In a study using transgenic mice, Rajji et al. were able to temporally manipulate the gene for NR1 subunit of the NMDA receptor in CA3 of the hippocampus. The CA3 NR1-deleted mice were impaired in learning that digging response to a cup scented with odor A, for example, was rewarded in context 1, but not in context 2, and *vice versa* for odor B. This was the case when scented objects (cups with scented sand) and contexts were novel, but not for familiar odors and contexts. The perceptual discrimination of contexts or items by themselves was not impaired in the NR1-deleted mice. Komorowski et al. also used the same paradigm in rats and found the neural correlates for representing the item–context paired associations in the hippocampus (Komorowski et al., 2009). The dentate gyrus (DG) appears to be also critical (presumably in conjunction with CA3) in this contextual odor-selection task because selective neurotoxic lesions in DG in the dorsal hippocampus also disrupted the acquisition of the task (Morris et al., 2013).

**Figure 7:**
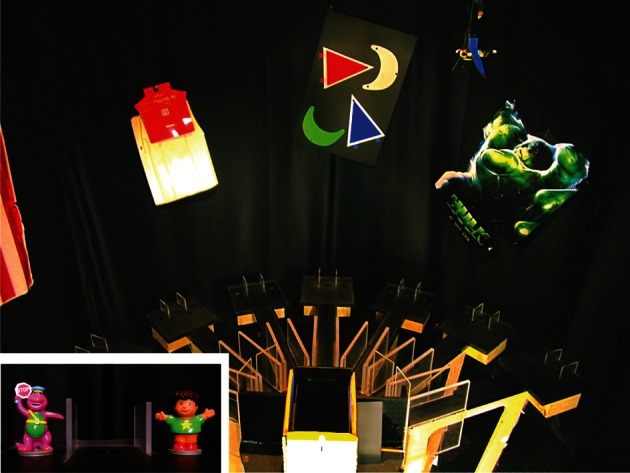
**A contextual item-selection task.** The task requires the rat to choose one of the objects (inset: toy figures) depending on where in the maze the two objects appear. The surrounding visual cues on the curtains serve as a visual context.

In our laboratory, we have verified that the hippocampus, prefrontal cortex (PFC), and PER play critical roles and interact with each other as rats are engaged in contextual item-selection behavior. Specifically, we developed a behavioral paradigm in which different arms of a radial-arm maze were associated with either an object-in-context rule or a response-in-context rule in order to test goal-directed behavior (Lee and Solivan, 2008). Rats were required to make an explicit choice of an object associated with reward in a given context (i.e., arm-associated visual cues in the background). Specifically, a pair of different toy objects was presented in one of the four arms in a large radial-arm maze with seven arms (Figure 7). The two objects were placed on top of food wells in a small rectangular platform at the end of a given arm and the rat was given only a single opportunity for exposing one of the food wells covered by the objects (by displacing the object with its front paw or snout). The configuration of placing the two objects in a given arm varied from trial to trial. Two of the arms were associated with the object-in-context rule and, when the rat entered the object-in-context arms, object identity information was a critical factor because the rat was required to ignore the food-well locations associated with the objects and should displace a particular object in that context regardless of whether the object appeared on its left or right side. If the rat entered one of the two arms associated with the response-in-context rule, the rat should ignore object identity because displacing any object occupying the food well on a particular side of the rat (or over a particular food well) was rewarded in a given context. The design of the task thus required the rat to pay attention to (a) the arm-associated background context for a given trial, (b) the strategy or rule that was relevant in the context, and (c) specific object or response information associated with reward in a given context for obtaining reward.

In our task, since the maze was surrounded by rich visual cues in the background, rats must pay attention to the visual context in order to choose a contextually correct object or response. It is unlikely that rats were dependent more on path integration in identifying the arms visited because, once exiting the start box, rats usually explored the center stage (frequently showing rearing behavior) before finding and entering an open arm. Despite the seemingly complex task demands with potentially conflicting strategies (i.e., object-in-context vs. response-in-context) associated with different contexts, normal rats pre-trained to displace an object to retrieve a food reward learned the task only in approximately 1 week on average. The learning curve itself is interesting because of its non-linear property. That is, rats typically showed almost 50% chance-level performances for several days (approximately 6–7 days) but suddenly exhibited a jump in performance at around day 7 or so during the acquisition. Such learning curve suggests that the rat experienced an “a-ha” moment suddenly after several days of having tried to figure out what the task demands were. This is likely because a typical source of poor performance during acquisition was the *response bias* erroneously associated with object-in-context arms. For example, when a rat happened to displace a correct object (e.g., object A) on the left food well in arm 3 (one of the object-in-context arms) at an early learning stage, the rat appeared to become strongly inclined to adopt a response strategy almost immediately. That is, the rat seemed to make an erroneous association and might “think” that it obtained reward because it turned to the object on its left side in the context (instead of associating the object identity with the context and reward). It appears that this dominance of response bias over object-in-context strategy is an innate bias that all rats show almost without exception, albeit to different degrees among individual rats. During the earlier phase of learning, rats acted as if they were only governed by the response-in-context strategy but they started to show *inhibitory behavior* immediately in front of a wrong object after a few days of training, which was usually a sure sign that foretold an upcoming performance surge in the task in 2–3 days.

Rats with axon-sparing excitotoxic lesions in the hippocampus were severely impaired in the task described above and their performance stayed almost at chance level throughout 10 days of post-surgical testing (Lee and Solivan, 2008), suggesting that normal hippocampal function was required in the task. Interestingly, the performance deficits were more severe in object-in-context trials than in response-in-context trials with hippocampal lesions. That is, the lesioned rats showed gradual improvement in performance in response-in-context trials, whereas the same rats exhibited chance-level performances throughout 10 days of testing in object-in-context trials. The severe performance deficits in the object-in-context arms was not attributable to impairment in object recognition because the hippocampal lesioned rats were normal in discriminating objects when only object recognition was required without any demand for contextual information processing. Overall, it is important to note that the hippocampal lesioned rats were normal in simple object discrimination and also gradually improved when no contextual object information processing was required (i.e., in response-in-context arms), but showed severe and irreversible impairment only in object-in-context arms where processing object information in the specific arm context was critical.

In our contextual object memory task (Lee and Solivan, 2008), the mPFC was also manipulated with MUS in a separate group of rats. Inactivations of the mPFC with MUS dropped the performance in response-in-context trials down to approximately 80% (as compared to almost perfect performance with saline injections) on the first day of MUS injection, but the rats still performed the task reasonably well. On the next day, however, the same drug did not affect the response-in-context performance any more. In contrast, the MUS inactivations of the mPFC caused more severe deficits in object-in-context conditions (experienced in the same experimental session with the response-in-context conditions), producing almost chance-level performances throughout 2 days of MUS conditions with no sign of recovery. The more pronounced deficits in the object-in-context type of trials than in the response-in-context type of trials suggest that mPFC is necessary for normally disambiguating object identities using background contextual information in rats. As demonstrated in the response-in-context trials, the mPFC-inactivated rats were relatively normal in strategically showing correct responses (turning to left or right food well) according to the context associated with a visiting arm and this suggests strongly that the severe deficits of those rats in object-in-context trials were not merely due to inabilities of those rats to remember a context-associated rule or to inhibit inappropriate responses in the task.

In order to find the neural correlates of contextual object selection, we recorded multiple single units simultaneously from the hippocampus and mPFC using the same task. In the unit recording study, neural firing was recorded throughout the acquisition period until the rats showed asymptotic performances in order to find neural correlates of task acquisition. Because of the clearly observable jump in performance during the acquisition (at around day 7), it was not difficult to draw a boundary between pre-learning and post-learning stages in the task. Hippocampal neurons mostly fired in a spatially localized fashion (e.g., arm-specific place fields) as reported in prior studies (O'Keefe, 1976; O'Keefe and Nadel, 1978; Muller et al., 1987). As has been also reported previously (Breese et al., 1989; Markus et al., 1995; Kobayashi et al., 1997; Lee et al., 2006), cells heavily represented the regions where reward-associated events took place. Object information alone, however, did not significantly explain the modulation of neuronal firing. This could result from the possibility that, within the hippocampus, object information is not represented by itself but always in conjunction with contextual information (Komorowski et al., 2009; Navawongse and Eichenbaum, 2013). Interestingly, there was a transition in the firing properties of neurons in the hippocampus across learning stages. As noted in the perturbation studies (Lee and Solivan, 2008, 2010; Jo and Lee, 2010a,b), rats first learned to choose objects using the contextual response strategy (e.g., choose any object on the left side in arm 3) at the earlier stage of acquisition in this task. It was more likely to observe the neurons that fired at higher rates in the trials in which the same turning responses were observed in a given context (regardless of object identities) during the pre-learning period than in the post-learning period. As the rats learned the task and the performance increased during learning, the proportion of neurons whose firing patterns were modulated by the response-in-context strategy decreased significantly and more neurons started to fire at higher rates in their preferred firing locations when the rat was about to push the *contextually correct object* irrespective of the turning directions associated with those choices (Lee and Kim, 2010; Kim et al., 2011). It is known that complex spike neurons (often characterized as place cells) in the hippocampus modulate firing rates within their preferred firing locations (i.e., place fields) in response to subtle yet significant changes in the animal's external and/or internal environment (Hetherington and Shapiro, 1997; Wood et al., 2000; Lee et al., 2006; Leutgeb et al., 2007). Most of these unit-recording studies were conducted in foraging situations in which the animal freely moved around in all directions in order to collect scattered rewards. The results from our study strongly suggest that, in a goal-directed task, the within-field firing-rate modulation in the hippocampus is likely to be a physiological manifestation of hippocampal networks interacting with other brain regions (e.g., PFC) as spatial context is utilized according to other task-related variables such as objects and cognitive strategies.

Similar changes in the firing patterns of neurons in the mPFC across learning support the above conjecture. That is, during the initial learning of the task, the firing patterns of neurons in mPFC were critically modulated by the acquisition of the rule. Specifically, the rats used in the above study were implanted with a multi-electrode recording drive that could record single units (as well as local field potentials) from both hippocampus and mPFC simultaneously (Kim et al., 2011). Although the neurons in mPFC were not as spatially selective in firing (i.e., arm-specific firing) as those in the hippocampus, mPFC neurons also showed the strategy-dependent similarity in spatial firing patterns across learning as hippocampal neurons did. The increases in object-in-context-compatible firing patterns in both mPFC and hippocampus were significantly correlated with performance, suggesting that the mPFC-hippocampal areas work as a unified functional network for learning how to disambiguate objects using proper strategy and context information. Also in support of such reasoning, when pre-learning and post-learning periods were compared, more neurons in both mPFC and hippocampus synchronized their spiking timing with theta rhythms (the same region as well as from the other region) after the learning occurred only when the rats showed object-in-context strategy-compatible responses, but not when the same rats showed response-in-context-based responses. The proportions of neurons showing significant phase-locking to theta rhythms during response-in-context trials did not change, however, between the pre-learning and post-learning stages (Kim et al., 2011). A recent study has also shown that mPFC inactivations with MUS disrupt task demand-related firing patterns in the hippocampus in contextual item selection (Navawongse and Eichenbaum, 2013).

Interestingly, the mPFC neurons were distinguishable from the hippocampal neurons because more global task demands were coded in neuronal firing patterns in the mPFC but not in the hippocampus (Kim et al., 2011). That is, mPFC neurons fired similarly when the rat experienced the same type(s) of events between different contexts. For example, more neurons in mPFC (than in CA1 of the hippocampus) changed their firing patterns significantly when the rat entered an arm and/or chose an object regardless of *where* those events took place. Interestingly, such specificity in firing associated with a specific event type in mPFC neurons became more pronounced as learning progressed and were correlated with performance of the animals, whereas no such changes were found in CA1 neurons. The results suggest that the global task structure (e.g., “when the door opens, *go out* and *enter an open arm* and *push the correct object* to *get reward*”) is represented more prominently in the mPFC (Jung et al., 1998) than in the hippocampus. Although the electrophysiological correlates of mPFC neurons in the contextual memory task suggest that the neural firing in that region is modulated by multiple cognitive factors, it appears that the most critical task demand for recruiting mPFC is to require animals to choose between discrete responses to an object with ambiguous meanings (with respect to reward value) by using the visual context in the background (Lee and Shin, 2012).

What functions of the hippocampus would make the structure so critical for processing contextual item information? The contextual object-memory task described above required the rats first to identify the visual context of the environment associated with a visiting arm because the contextual information was used for determining the correct strategy and/or correct object information in that context. The literature suggests that the hippocampus is essential for either dissociating or generalizing similar contextual representations (Marr, 1971; McNaughton and Morris, 1987; O'Reilly and McClelland, 1994; Guzowski et al., 2004; Lee et al., 2004b; Leutgeb et al., 2004; Vazdarjanova and Guzowski, 2004). Dissociating similar neural representations is often called “pattern separation” or “orthogonalization” and the hippocampus appears to possess anatomically sufficient networks for decreasing overlap among memory representations. This function of the hippocampus may be critical in representing similar events into unique event memories. In contrast, it is believed that the hippocampal networks also perform a seemingly opposite computational function and it is called “pattern completion” or “generalization” of neural representations. It is highly likely that both pattern separation and completion processes were needed for identifying arm-associated contexts properly in our contextual object-memory task. Computational models have emphasized the functions of the DG and CA3 subfields in the hippocampus for pattern separation and completion processes. Detailed reviews on this topic can be found in other articles (Marr, 1971; McNaughton and Morris, 1987; O'Reilly and McClelland, 1994; Rolls and Treves, 1998; Guzowski et al., 2004). Briefly, it has been suggested that both sparse connectivity between the DG and CA3 via the mossy fiber pathway and the autoassociative network using massive recurrent collaterals within CA3 provide mechanisms with which both computational processes can be performed dynamically. If the suggested computational processes are required in our contextual memory task described above, it is predicted that lesions produced in DG should disrupt the performance in the task because contextual discrimination should suffer accordingly. So we tested if DG was necessary in the contextual object memory task mentioned above. The two arms used in the task were spaced relatively close to each other in order to create a situation where DG's pattern separation became essential (but not too close to establish good baseline performance). As computational models have suggested, DG-lesioned rats showed no sign of improvement in performance from chance level throughout 6 days of post-surgical testing, resulting in virtually the same results with those observed in total hippocampal lesioned rats. When the rats were tested with a wider separation between the contexts associated with the objects, DG-lesioned rats improved performance across days as opposed to the sustained deficits observed in the original condition presumably because less pattern separation was required with the wider arm separation. The results strongly suggest that the deficits in the hippocampal lesioned rats in the original contextual task were largely attributable to the loss of DG-CA3 networks although this needs to be verified with studies involving other subfield (e.g., CA3, CA1) lesions in the hippocampus. To our knowledge, this is the first experimental evidence showing that DG is necessary for contextual object information processing in a goal-directed task (Morris et al., 2013).

#### Primate and human studies

A surprisingly small number of studies have been conducted if only goal-directed memory tasks (but not spontaneous exploration paradigms) are reviewed with the topic of contextual item selection in primates. Constraining the scope of review using the definition of context of this review further reduces the number of studies available for review because this type of context is difficult to implement in primate studies. Specifically, monkeys are often tested using a Wisconsin General Testing Apparatus (Parkinson et al., 1988; Angeli et al., 1993; Malkova and Mishkin, 2003; Belcher et al., 2006) or computer monitors (Cahusac et al., 1989; Rolls et al., 1989; Rao et al., 1997; Suzuki et al., 1997; Dore et al., 1998; Charles et al., 2004; Baxter et al., 2007; Bachevalier and Nemanic, 2008; Wirth et al., 2009), and although item-place associations might be tested in those settings, the visual context in the background was often made irrelevant to the task (by darkening the background area). Place information associated with an object was in this case conveyed by food-well locations in a tray in front of the monkey's cage or by locations within a small computer monitor. In such experimental conditions, it is reasonable to assume that locations of objects were mostly identified using an egocentric frame of reference (i.e., with respect to the animal's body).

The nature of stimuli used in Gaffan group's studies (i.e., object-in-place scene memory task) may qualify those as proper studies to be reviewed here. That is, an object in the Gaffan's paradigm was a small typographic character that can be touched by the monkey in a computer monitor. A context was a visual image covering the entire computer screen and served as the background of the typographic objects. The background image (i.e., “scene” according to the Gaffan group) was composed of a random number of ellipses and segments of ellipses. The colors, positions, and sizes of elliptical shapes were randomly determined by a computer algorithm. The monkey was required to learn where in the monitor a certain typographic object was rewarded when a particular scene was presented as a contextual background. The Gaffan group showed that lesions produced in the fimbria-fornix (i.e., a fiber bundle that connects the hippocampus to subcortical regions such as mammillary bodies and thalamus), entorhinal cortex (EC), and orbital PFC resulted in learning deficits in the task (Gaffan, 1994; Charles et al., 2004; Baxter et al., 2007). Disconnecting the interactions between the fimbria-fornix and the PER also produced impairment (Gaffan and Parker, 1996). The operational definition of the term context in this review emphasized the role of context in removing critical ambiguity in target stimuli (e.g., objects) during choice behavior. Judging from that perspective, the background image used in the object-in-place scene memory task by the Gaffan group might have been used as a disambiguating cue. That is, by providing configural spatial information between the target object and other background elliptical shapes, the background image was also used as a spatial cue for helping the rats to determine whether the target character was in the right place or not for the purpose of obtaining reward. For the current review, however, it is arguably difficult to differentiate foreground objects from the background stimuli in this type of settings because the elliptical shapes contained in a scene may well be perceived as objects also.

In humans, only very few studies examined contextual item selection as opposed to the more frequent usage of the contextual response-selection paradigm as described above. Among those, Burgess et al. (2001) required subjects to navigate in a VR town. While navigating the town, the subject encountered various objects in different visual contexts (e.g., rooms) and was later required to choose a correct item when cued by one of the visual contexts. Comparing the contextual item-selection task with a non-contextual version revealed activations in the precuneus, parahippocampal cortex, retrosplenial cortex, and hippocampus. Other areas such as the posterior parietal cortex, cingulate cortex, and the prefrontal cortices also showed significant activations. In a study done by Hayes et al. (2004), one of the tasks (“spatial trial-landmark consistent” version) assessed whether a subject could recognize an item that appeared with a particular visual context in a previously watched movie film. Although there appears to be some confounds between visual contextual manipulation and object manipulation in this study (along with a temporal sequence factor), when compared to other object/temporal sequence related tasks, the contextual item-selection task activated the parahippocampal gyrus and PFC more significantly. A human-patient study (Hannula and Ranganath, 2008) also used a contextual item-selection paradigm. In the second experiment of the Hannula et al. study, hippocampal lesioned subjects saw a series of images that contained different items (faces in this case) overlaid on top of various visual contexts (natural scenes). After various time lags, subjects were required to recognize the face that had been previously associated with the current scene when that face was presented with other faces overlaid with the cueing scene. Hippocampal amnesic patients showed deficits in this contextual item-selection task irrespective of the time lags.

### Contextual item–response selection

So far, we have categorized contextual behaviors into two categories and those were contextual response-selection behavior and contextual item-selection behavior. There is another type of behavioral tasks in which these two categories were used in a mixed fashion. The need for this category of contextual behavior is related to the type of behavioral task in which only a single stimulus (e.g., object, tone, light) out of multiple candidate items is presented in a given context per trial and the animal is required to either respond to the stimulus directly (or to a dedicated response unit in the apparatus) or to inhibit the response. So it is similar to contextual response selection, but is different at the same time because different items need to be recognized.

#### Rodent studies

For example, Good and Honey used a behavioral paradigm in which the rat was trained to press a lever in response to either tone or light stimulus. Each stimulus, however, resulted in reward only in one context, but not in the other context (Good and Honey, 1991). Normal rats were able to emit the lever-pressing responses at different frequencies according to the stimulus–context paired associations, whereas the hippocampal-lesioned rats were impaired in doing so. In this paradigm, the same response should be selectively controlled (thus response selection involved) in a contextual fashion but also item information should be monitored in a discriminative fashion. Compared to contextual item-selection paradigms mentioned above, the response in this case was not targeted to an item itself but to a designated response lever. This situation is more akin to the representative example Hirsh used when he described how the hippocampal contextual memory influenced the stimulus-response “performance line” during the retrieval of associative information (Hirsh, 1974).

Gilbert and Kesner did a series of experiments on this topic (Gilbert and Kesner, 2002, 2003, 2004). In their typical behavioral paradigm, a rat exited a start box located in an area within a circular open field (i.e., cheeseboard maze) and had to choose whether to approach and displace (thus response selection between Go and No-Go responses) an object located in a certain place (or context) in the arena or not. Two different objects were used in the experiment and only one of the objects appeared in a given trial. The object appeared in one of the two fixed locations in the circular field and each object contained a reward underneath it only when it appeared in a particular location. In this task, as the start box door was opened, the rat saw an object against a certain visual background in the room because the open field was not walled off from the environment. This task thus required contextual item–response selection. Normal rats learned to approach the rewarding object and to inhibit such responses to the unrewarded object, whereas electrolytic lesions in the hippocampus completely abolished such learning capability (Gilbert and Kesner, 2002) as well as performance when lesions were produced after learning (Gilbert and Kesner, 2004). The same results were reported when different odors must be discriminated contextually (but not when object-odor associations were required). However, the hippocampal lesioned rats were normal in discriminating objects, odors, and places when tested separately without any associative demand. The Kesner group also showed that CA3 in the dorsal hippocampus was critical in learning the task (Gilbert and Kesner, 2003) presumably attributable to the autoassociative network in that region (O'Reilly and McClelland, 1994; McClelland and Goddard, 1996; Rolls and Kesner, 2006).

The perturbation studies mentioned so far in this review mostly tested *recognition* of contextual item representations because animals were presented with objects in certain contexts and they were required to respond to the object-context conjunctions according to task demands. Some goal-directed tasks, however, required *cued recall* in similar settings (Day et al., 2003; Tse et al., 2007; Kesner et al., 2008). For example, in the Day et al. (2003) study, rats were required to learn flavor-place paired association during a sample phase in a large open field. In the following test phase, the animal was cued with the flavor that had been sampled previously in a remote location (i.e., start box) and was required to revisit the original paired-associate place on the basis of the olfactory cue sampled. It was shown that the NMDA receptor-mediated plasticity mechanisms in the hippocampus were critical for normal learning to occur in the task. Kesner et al. (2008) also used a similar paradigm (but with objects instead of odors) and reported the importance of CA3 in the hippocampus. The above paradigms, however, test more than just contextual object information processing because the tasks required the animals to “hold” the cue information (e.g., flavor or object) sampled in the start box in working memory during the navigational search for the paired associate location after they exited the start box. Furthermore, the tasks relied on intact navigational capability of rats in open space. In other words, even if the rat recalled the paired associate location at the time of being cued by the cueing odor in the start box, it could still produce performance deficits if normal spatial navigation that should guide the rat to the paired associate location was impaired as a result of hippocampal perturbations.

**Figure 8:**
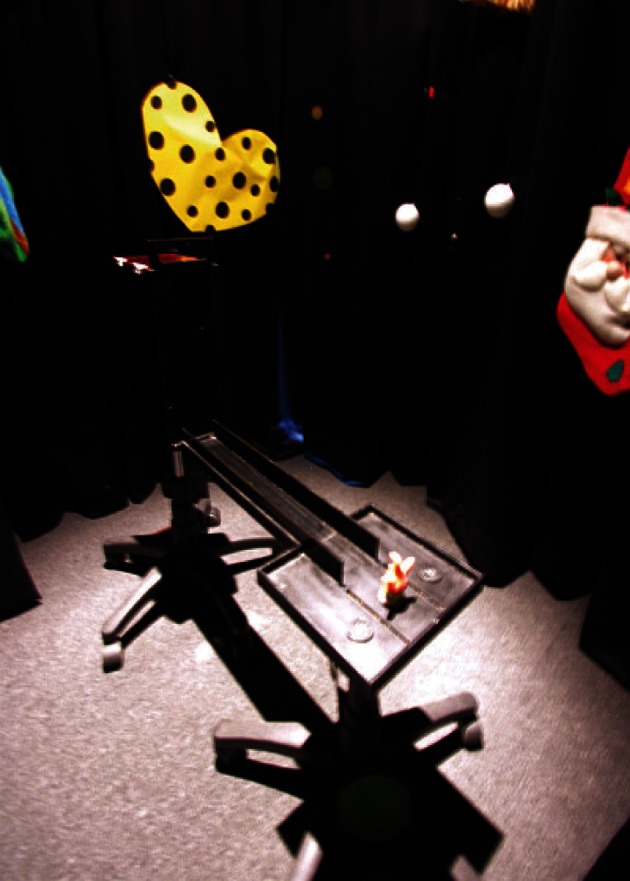
**An object-cued response selection task.** The rat exits the start box and runs along the linear track and encounters an object cue (orange toy figure in this example). The object signals which disc on its left or right side should be displaced for successful retrieval of food. Rats are impaired in this task when the distal visual cues in the background are removed (by darkening the room).

We tested recently whether those additional cognitive processes mentioned above were critical in testing hippocampal functions for contextual item–response selection by using a simpler paradigm (Yoon et al., 2012). In our task, the rat just ran along a straight track (thus with almost no requirement for spatial navigation) upon exiting a start box and encountered a toy object at the end of the track (Figure 8). The rat was required to simply choose between the two food wells (covered with identical metal discs) on both sides of the cueing object. Two objects were repeatedly used across trials with one of the objects cued the presence of reward in the left food well and the other object conveyed the opposite information. The track was located in a circular curtained area and the curtains were decorated with distinct visual cues (brightly illuminated with halogen lights) to maximally encourage the rats to use visual contexts in the background. The experimental design thus did not require spatial working memory because the paired reward location was found adjacent to the cueing object and the track guided the rat to the object. Although one may predict that this task may have been solved easily by associating egocentric responses with cueing objects (which is not known to be hippocampal dependent), surprisingly, inactivation of the dorsal hippocampus with muscimol severely disrupted normal performance for two consecutive days. We confirmed that the rats used visual cues in the background by showing that rats were not able to do this task in the dark either with or without hippocampal inactivations (Yoon et al., 2012). We also showed that rats were able to perceptually discriminate the cueing objects in the dark (presumably using tactile information). This task thus meets the requirement of contextual item–response selection because two different responses (left and right turns) must be associated with two different items (but single item per trial) for reward. Although it can be considered also as a variant of contextual response selection because it was more of an item-cued contextual response selection, rats had no problem in discriminating the cueing objects as well as food-well location by themselves with hippocampal inactivations. The results strongly suggest that retrieving object-context conjunctive representations requires the hippocampus even when additional cognitive demands were not imposed in a goal-directed task.

#### Primate and human studies

In the study by the Rolls et al. (2005), monkeys were required to associate object-displaying monitors with local room contexts. The positions of both monkey and monitors were varied across trials but arrangements were made in such a way that the monkey could view certain room cues constantly in association with a particular monitor. There were two monitors in the room and the monkey should lick a sipping tube for obtaining reward when object image A, for example, appeared in one monitor, but should avoid licking the tube (to avoid saline solution) when the same object image appeared in the other monitor. Another object image B appeared always with object A but with opposite reward contingencies (thus making the task a biconditional object-place paired associate task). This task thus requires response selection in conjunction with contextual item information. Object-associated ambiguity was maximal in the task because the same objects were used as both positive and negative stimuli depending on the background room context in which they were presented. It is important to note that, unlike the Gaffan group's scene memory task, the monitor itself did not contain any background image (only an object cue was shown), thus allowing more straightforward definitions for objects and contexts in the task. The Rolls et al. study is exceptional as compared to other primate studies because object images in computer monitors were associated with actual room cues in the background as in rodent behavioral studies. Rolls et al. verified the dominant usage of the room context (but not egocentric reference frame) in the task by recording “spatial view cells” that responded equally to the monitor positions even when the monkey's egocentric relationships with the monitors changed. Neurons in the hippocampus and PER responded to object identity, the background context in which they appear (i.e., place), and the combination of those two factors according to the study. It is reported in the study that firing patterns of only approximately 10% of neurons were significantly modulated by object information both in the hippocampus and the PER, which is somewhat surprising because the PER is considered as the major area for representing object identity information in the medial temporal lobe. What is more interesting in this study, given that the PER is considered for processing non-spatial information by many researchers, is that approximately equal proportions of neurons in the PER responded significantly to place information as well as to object-place paired associative information. Rolls et al. emphasized that the results might be attributable to the recording locations in the brain being at the boundaries between the posterior PER and parahippocampal cortex. However, it is also possible that the results might reflect the actual functional firing properties of PER neurons in contextual object information processing. Except for the Rolls et al. study, almost all studies using primates used local positions within a computer monitor or within a testing tray when investigating object-place associations.

## Neural circuits for contextual behavior

**Figure 9:**
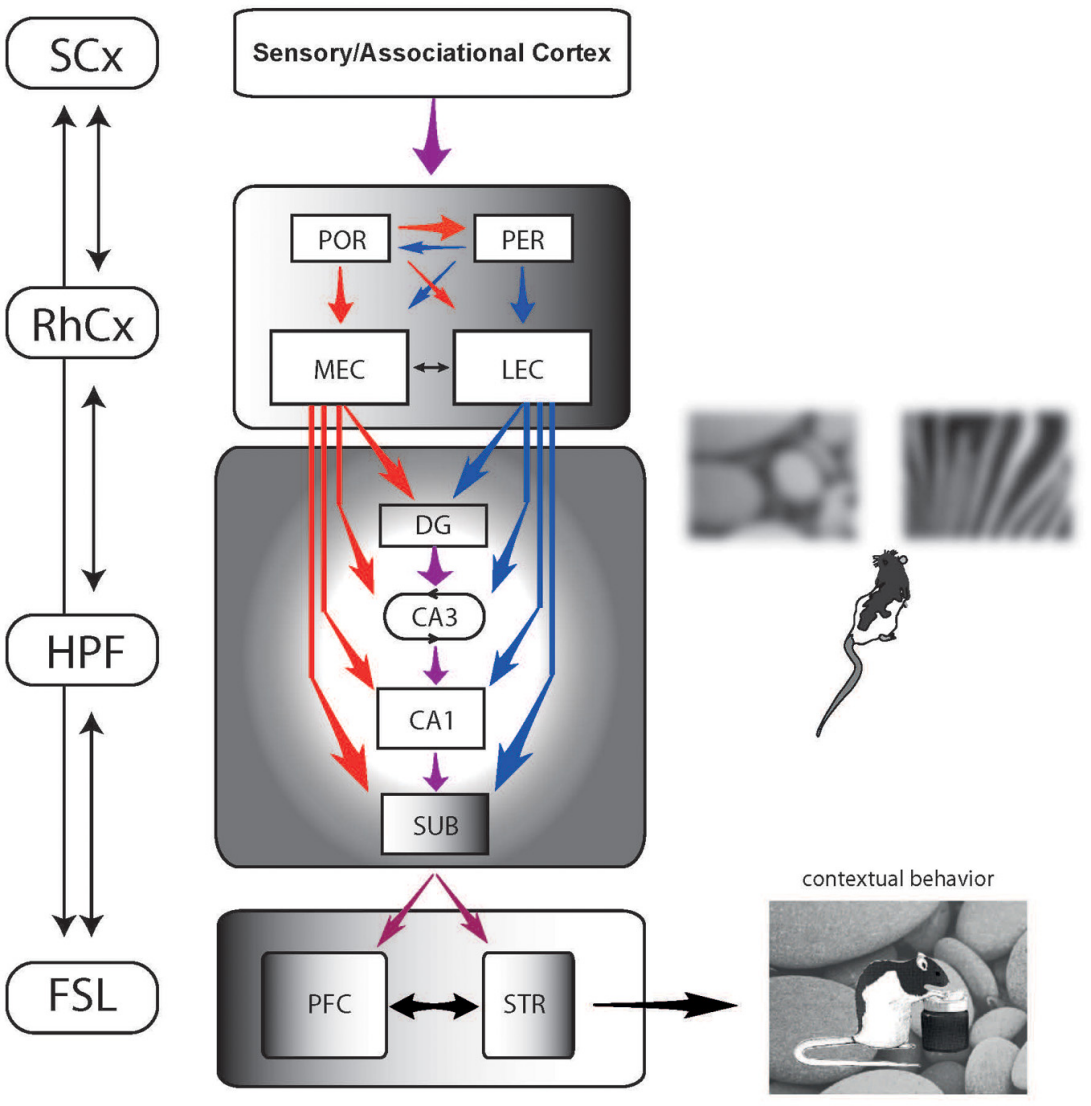
**A schematic illustration of the information flow and interactions among the brain regions (selectively chosen) involved in contextual behavior (connections are simplified for illustrative purposes).** Via primary and higher-order sensory cortices (SCx), multimodal perceptual information from the environment enters the rhinal cortical regions (RhCx) that include the post-rhinal cortex (POR), perirhinal cortex (PER), medial entorhinal cortex (MEC), and lateral entorhinal cortex (LEC). The areas in the RhCx may interact with each other to varying degrees as indicated by arrows. It is hypothesized that qualitatively different information-processing streams exist in the RhCx, denoted by red arrows (contextual information) and blue arrows (item information) here. The qualitatively different information streams continue as the information enters the hippocampal formation (HPF) in which associative processes occur between these two streams (presumably in order to form an event representation). The hippocampal subfields (DG, CA3, CA1) and subiculum (SUB) all receive contextual-noncontextual information in parallel. Serial information processing across the hippocampal subfields to SUB also occurs at the same time. The DG-CA3 network (circular arrow indicating the recurrent network in CA3) is particularly important for recognizing ambiguous/modified contexts in comparison to memory representations (e.g., rat discriminating different visual contexts modified from the original ones). The information regarding the contextual interpretation of the environment and its associated items then interacts with the fronto-striatal networks (FSL) including the prefrontal cortex (PFC) and striatum (STR) in a goal-directed manner before final response behavior is determined (e.g., digging in pebble context here). The four regions (SCx, RhCx, HPF, and FSL) interact with each other via various feedforward and feedback connections to realize coherent, inter-regional bottom-up and top-down communications toward goal-directed behavior.

Currently, an influential theory (Fyhn et al., 2004; Knierim et al., 2006; Furtak et al., 2007; Kerr et al., 2007; Eichenbaum and Lipton, 2008) posits that contextual background stimuli reviewed above are represented in the hippocampal formation (hippocampus plus subiculum; HPF in Figure 9) via the rhinal cortical regions (RhCx in Figure 9) associated with the hippocampus, including the PER, postrhinal cortex (POR), and EC. Although the circuits and the types of information processed are often discussed from the viewpoint of spatial–nonspatial information processing for memory, we would argue that the same circuits are involved in processing background contextual information (red arrows in Figure 9) and item information within the context (blue arrows in Figure 9). Some coarse dissociations have emerged among the RhCx regions regarding the representation of spatial vs. non-spatial information (Fyhn et al., 2004; Hargreaves et al., 2005; Knierim et al., 2006; Brun et al., 2008; Deshmukh and Knierim, 2011; Deshmukh et al., 2012). That is, the PER and lateral EC (LEC) carry less spatial information than the POR and medial EC (MEC). However, it is still largely unknown how exactly these neural circuits work together to influence the animal's behavior in a contextual manner because most of the physiological studies were performed using non-mnemonic tasks in which no goal-directed behavioral selection was necessary. As shown in Figure 9, contextual and item information should influence the hippocampal subfields and subiculum both serially (via the trisynaptic circuits from DG to CA1) and in parallel (via perforant paths independently synapsing onto all three subfields). Computations that occur in the hippocampal subfields have been experimentally investigated explosively in the last 10 years although most studies heavily focused on spatial contextual representation (Jung and McNaughton, 1993; Kesner et al., 2000, 2004; Nakazawa et al., 2002; Lee et al., 2004a,b; Leutgeb et al., 2004, 2005; Vazdarjanova and Guzowski, 2004; Rajji et al., 2006; McHugh et al., 2007; McHugh and Tonegawa, 2009; Jones and McHugh, 2011; Yassa and Stark, 2011; Nakashiba et al., 2012). Apparently, CA3 (with the conjoint effort of DG) plays key roles in recognizing the original as well as modified contextual environments (Marr, 1971; Treves and Rolls, 1992; Rolls, 1996). It would be rare in natural settings to encounter the same context in exactly the same physical conditions every time an animal experiences the context repeatedly (due to differences in lighting conditions, viewpoint, degradation or loss of some elements in the context, etc.). For an animal to use contextual memory and learned contextual behavior, however, it is critical to reliably identify the same context despite some minor changes in its surrounding as well as to recognize some significant differences. The hippocampal circuits appear to perform such critical computations. The CA3 subfield, for example, contains autoassociative networks whereby pattern completion recovers the learned contextual representation despite some modification/loss/noise in the original context (Marr, 1971; McNaughton and Morris, 1987; O'Reilly and McClelland, 1994; Kesner et al., 2000; Lee et al., 2004b; Rolls and Kesner, 2006). The same network appears to perform non-linear operations to orthogonalize similar, yet significantly different contextual information into a separate contextual memory (pattern separation) and the DG subfield seems also critical during these processes (O'Reilly and McClelland, 1994; Kesner et al., 2000; Leutgeb et al., 2007). Considering that one of the important points of this review is to direct the attention to the importance of understanding the contextual information processing in connection to the animal's behavioral output, it is worth mentioning that some critical issues still remain unresolved in the hippocampal research field. Most of all, it still needs to be understood why CA1 does not normally show CA3-like non-linear representational dynamics (between pattern-completed and pattern-separated states). Some dissociations existing in the literature between CA1 and CA3 even make this issue more interesting (Gilbert and Kesner, 2003; Kesner et al., 2004; Lee and Kesner, 2004; Lee et al., 2005; Hoge and Kesner, 2007). Since the brain structures such as the PFC and STR that may influence the final behavioral selection or choice more directly receive direct inputs from the CA1 subfield and the subiculum, but not from DG and CA3, it still needs to be found how the dynamic representational changes in DG-CA3 circuits influence the final selection behavior of the animal in a goal-directed contextual memory task. That is, what is the functional significance of the hippocampal subfield computations? It also needs to be determined what the subiculum exactly do in contextual information processing and the resulting behavior? Considering that cortical outputs of the hippocampus are relayed via the subiculum, more efforts should be made to uncover how the hippocampal information processing influences its downstream structures in contextual behavior.

The hippocampal subfields should pass the most powerful contextual representation to various networks in the brain (based on the research findings so far) and this may the most important source of information the brain uses to set the mode of operation in the face of a specific context. The contextual information from the hippocampus should be useful particularly for those brain areas whose functions are critical for controlling final action or response. The PFC and the STR have long been suggested as key players in this regard. For example, many studies have shown the roles of PFC in flexible choice behavior (Ragozzino et al., 1999; Rich and Shapiro, 2007; Roy et al., 2010; Zeithamova and Preston, 2010; Lee and Shin, 2012) and resolving conflicts in response (Badre and Wagner, 2004; Haddon and Killcross, 2006; Marquis et al., 2007; Oualian and Gisquet-Verrier, 2010; Horga et al., 2011). Contextual behavior presumes that multiple behavioral responses should be put out conditionally as different environments are experienced. Therefore, flexible behavioral control in a contextual manner should be essential and it is not surprising that PFC is a critical part of contextual behavior in that sense. Since it has long been shown that PFC does work as a flexible functional integrator for other domains that are not necessarily by themselves contextual (Tomita et al., 1999; Miller, 2000; Miller et al., 2002), it would be inappropriate to make a statement that PFC is dedicated for contextual behavior. It is possible that PFC possesses some capability of influencing how contextual information is processed in the brain, given the multimodal anatomical connections it maintains with other cortical and subcortical regions including the hippocampus (Ranganath et al., 2004; Haddon and Killcross, 2007; Jo et al., 2007; Marquis et al., 2007; Horga et al., 2011; Euston et al., 2012; Navawongse and Eichenbaum, 2013). It still needs to be determined what the major differences are between the hippocampal network and PFC network in terms of handling contextual information. For example, although the PFC network may maintain the contextual memory for a limited duration of time (e.g., several seconds), the hippocampal circuits are critical for maintaining contextual memory over a certain period of time exceeding the short-term range (e.g., several minutes) based on a previous study (Lee and Kesner, 2003). It remains to be seen whether there are other key differences between the two regions with respect to the types of computations for contextual information (e.g., pattern separation and completion).

The functions of STR and the cortico-striatal networks also have long been investigated (Hikosaka and Isoda, 2010; Ghahremani et al., 2012; Seo et al., 2012; Van Schouwenburg et al., 2012) with respect to how animals emit and control proper actions. As has been the case with the hippocampus and its subfields, the STR is considered as a complex brain region composed of heterogeneous functional subfields, for example, responsible for simple/rapid habitual stimulus–response association (McDonald and White, 1993; Packard and Knowlton, 2002; White and McDonald, 2002; Featherstone and McDonald, 2004, 2005), representation of value/motivation-related information (Yin et al., 2005; Scimeca and Badre, 2012; Stalnaker et al., 2012; Tai et al., 2012; Kim et al., 2013), flexible switching between learned behaviors and task demands (McDonald and White, 1993; Crofts et al., 2001; Cools et al., 2002; Ragozzino et al., 2002; Holahan et al., 2005; Eschenko and Mizumori, 2007; Braun and Hauber, 2011), to name a few. Since the dorsomedial STR (dmSTR) receives direct inputs from the mPFC (and direct visual cortical inputs) and the ventral STR (vSTR) receives hippocampal inputs (Siegel et al., 1975; Swanson and Cowan, 1977; McGeorge and Faull, 1989; Saint-Cyr et al., 1990; Berendse et al., 1992; Serizawa et al., 1994; Lopez-Figueroa et al., 1995; Voorn et al., 2004; Schulz et al., 2009), it is likely that the STR serves as a critical node for exerting critical influence on producing a final behavioral response in association with context. For example, a habitual behavior in response to a stimulus or item (presumably represented in the dorsolateral striatum, or dlSTR) may be contextually expressed via the functional connection between dmSTR and dlSTR. In fact, this may be the typical situation Hirsh (1974) used to illustrate how contextual memory by the hippocampus influenced the stimulus–response performance line (i.e., simple stimulus–response associative memory system) in the brain. Not many studies have studied the STR in a contextual memory task except for the ones for investigating the allocentric and egocentric navigations for the relative contributions of dmSTR and dlSTR between the two type of navigations (Whishaw et al., 1987; McDonald and White, 1994; Packard and McGaugh, 1996; Devan et al., 1999, 2011; Packard and Knowlton, 2002; Holahan et al., 2005; Mizumori et al., 2009; Brown et al., 2012; Lozano et al., 2013). Some physiological studies have been conducted (Mizumori et al., 2004; Thorn et al., 2010; Yamin et al., 2013), but more studies are needed to explore the less well-known aspects of STR functions in representing surrounding visual context and contextual behavior.

## Concluding remarks

In this review, efforts have been made both implicitly and explicitly to emphasize the importance of understanding contextual behavior with the consideration of final goal-related responses. For example, it is critical to understand how the contextual representation in the hippocampus is used by other regions in the brain such as the mPFC and STR in determining the animal's behavior in response to the context. Because the anatomy and physiology suggest that the circuits are built to influence the information processing among these and other contextual behavioral networks in both feedforward and feedback directions, investigating a particular neural structure in isolation may result in insufficient understanding of the network dynamics. As has been emphasized in this review, using goal-directed tasks with well-defined stimulus control is also critical during the investigative endeavor to answer the critical question of where in the information-processing stream the neural manifestation of context emerges as opposed to the representation of a mere sum of elemental percepts. This theme also echoes in understanding the neural systems that were previously considered as pure perceptual systems (Sobotka et al., 1997; Park and Lee, 2000; Poghosyan et al., 2005; Fenske et al., 2006; Rajkai et al., 2008; Chapuis and Wilson, 2012; Ley et al., 2012). These motivations led us to selectively categorize the existing literature including our own studies into discrete categories of contextual behavior mainly by using the criterion of how context, item, and response to those two components are interrelated. This scheme may have surely left out some other contextual behavioral tasks intentionally and unintentionally, but we reason that investigating contextual information processing and contextual behavior in an experimental setting with clearly defined physical stimuli and well-defined purposeful behavior should take priority over using other paradigms at this stage of neural investigation of contextual behavior.

### Conflict of interest statement

The authors declare that the research was conducted in the absence of any commercial or financial relationships that could be construed as a potential conflict of interest.
